# A Review on the Immunomodulatory Mechanism of Acupuncture in the Treatment of Inflammatory Bowel Disease

**DOI:** 10.1155/2022/8528938

**Published:** 2022-01-15

**Authors:** Zhifeng Liu, Yi Jiao, Tianyuan Yu, Hourong Wang, Yingqi Zhang, Di Liu, Yajing Xu, Qian Guan, Mengqian Lu

**Affiliations:** ^1^School of Acupuncture-Moxibustion and Tuina, Beijing University of Chinese Medicine, Beijing 102488, China; ^2^Acupuncture Department, Oriental Hospital of Beijing University of Chinese Medicine, Beijing 100078, China

## Abstract

Inflammatory bowel disease (IBD) is a chronic inflammatory disease with a high prevalence and canceration rate. The immune disorder is one of the recognized mechanisms. Acupuncture is widely used to treat patients with IBD. In recent years, an increasing number of studies have proven the effectiveness of acupuncture in the treatment of IBD, and some progress has been made in the mechanism. In this paper, we reviewed the studies related to acupuncture for IBD and focused on the immunomodulatory mechanism. We found that acupuncture could regulate the innate and adaptive immunity of IBD patients in many ways. Acupuncture exerts innate immunomodulatory effects by regulating intestinal epithelial barrier, toll-like receptors, NLRP3 inflammasomes, oxidative stress, and endoplasmic reticulum stress and exerts adaptive immunomodulation by regulating the balance of Th17/Treg and Th1/Th2 cells. In addition, acupuncture can also regulate intestinal flora.

## 1. Introduction

Inflammatory bowel disease (IBD) is a chronic, inflammatory, and autoimmune intestinal disease, which is characterized by abdominal pain, diarrhea, pus and bloody stool, intestinal obstruction, and intestinal perforation [1, 2]. IBD includes Crohn's disease (CD), a disease involving the whole digestive tract, and ulcerative colitis (UC), a disease only involving the colon [3]. IBD has become a global disease that poses a serious threat to human health worldwide with a high prevalence and canceration rate [4–6]. Epidemiological results show that the prevalence of IBD has been increasing in the past decade. The prevalence rate can be as high as 0.3% to 0.5% in Western countries such as Europe and North America and 0.05% in East Asia [7], with the canceration rate as high as 15% [8]. A cohort study published in 2020 showed that the risk ratios of colorectal cancer occurrence and death between CD patients and the general population were 1.4 and 1.74, respectively [9].

The pathogenesis of IBD is unclear and is generally believed to be the result of the interaction between the host immune system and gut microbiota, as well as genetic susceptibility and environmental susceptibility [10, 11]. The immune disorder is one of the recognized mechanisms, which plays an important role in the occurrence, development, and prognosis of IBD [12, 13]. Under the influence of environmental factors such as diet and smoking and the participation of intestinal flora, genetically susceptible people initiate an intestinal innate and adaptive immune response, resulting in intestinal mucosal barrier damage, ulcer, inflammatory cell infiltration, and other pathological changes.

Acupuncture, one of the most popular nonpharmacological therapies, has been used worldwide to treat patients with IBD due to its remarkable effect [14, 15]. It can not only improve the main symptoms of IBD patients, such as abdominal pain, diarrhea, and bloody purulent stool but also alleviate the accompanying symptoms, such as anxiety, depression, and fatigue [16–18]. A meta-analysis involving 13 RCTs with 1030 participants showed that acupuncture alone and acupuncture combined with medicine were more effective than conventional medicine in the treatment of UC [19]. Another meta-analysis showed that acupuncture and moxibustion were more effective than oral sulphasalazine in treating patients with IBD [18]. A single-blind randomized trial proved that electroacupuncture can reduce fatigue scores in IBD patients compared with those on the waitlist [17]. In recent years, the mechanism of acupuncture in the treatment of IBD has made certain progress, especially in the regulation of immune disorders. Therefore, this review summarized the previous studies to explain the immunomodulatory mechanism of acupuncture in the treatment of IBD.

## 2. Immune Disorder is One of the Recognized Mechanisms Leading to IBD

Intestinal immune function is exerted by innate immunity and adaptive immunity. It is believed that the disorder of mucosal barrier function is the main cause of IBD [20]. Antigen stimulates the damage of intestinal mucosa, increases mucosal permeability, stimulates the production of various inflammatory factors, and then makes the body participate in the adaptive immune response network. Therefore, both innate and adaptive immune disorders may lead to IBD.

### 2.1. Innate Immune System

Innate immunity is the body's first line of defense, which plays a vital role in identifying pathogens and maintaining the balance of the intestinal environment. In the early stage of intestinal inflammation, neutrophils infiltrate intestinal mucosa and epithelium, weaken the function of the epithelial barrier, destroy tissue structure, release proinflammatory factors, and enhance inflammatory response [21]. Immune cells such as macrophages, neutrophils, epithelial cells, and endothelial cells are involved in the intestinal innate immune response.

### 2.2. Adaptive Immune System

Under normal circumstances, the components of the adaptive immune system cooperate with each other and trigger an effective immune response with the molecules and cells of the innate immune system so as to eliminate invasive pathogens. The imbalanced expression of immune cells is one of the pathogenesis of IBD. CD4^+^T cells are important immune cells in the human body, and their abnormal activation, as an important mechanism, leads to intestinal mucosal immune response. CD4^+^T cells are divided into T regulatory (Treg) cells and T helper (Th) cells, among which Th cells are divided into Th1, Th2, Th17, and other subtypes. The imbalance of Th17/Treg and Th1/Th2 is the main cause of IBD.

## 3. Acupuncture Exerts Immunomodulatory Effects by Regulating the Innate System of the Intestinal Mucosa

### 3.1. Intestinal Epithelial Barrier

Intestinal epithelial cells and tight junctions between cells constitute the intestinal epithelial barrier. The morphological and functional damage of epithelial cells can lead to intestinal inflammation, and continuous inflammatory stimulation can lead to intestinal fibrosis [22]. Studies have shown that colonic collagen fibers of CD rats proliferate while the expression of collagen fibers decreases after electroacupuncture treatment, and the contents of hyaluronic acid (HA), procollagen III (PC III), and procollagen III (PC III) in serum decrease, indicating that electroacupuncture can improve the pathological state of intestinal fibrosis in CD rats [23]. Tumor necrosis factor-*α* is the key cytokine causing CD, which can induce intestinal epithelial apoptosis through its receptors (TNFR1 and TNFR2), resulting in intestinal epithelial barrier damage. Acupuncture combined with moxibustion can reduce the contents of TNF-*α*, TNFR1, and TNFR2 in the intestinal mucosa of CD patients [24, 25], and it can also reduce the apoptosis rate of intestinal epithelial cells [24].

**Figure 1:**
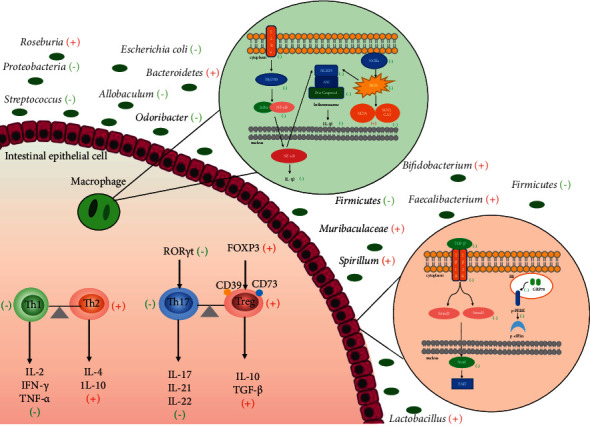
The mechanism underlying immune regulation of acupuncture. (+): upregulate; (−): downregulate.

Shi et al. [26] demonstrated that herbs-moxibustion combined with acupuncture at Zusanli (ST36) and Shangjuxu (ST37) could upregulate the expression of E-cadherin, the epithelial cell marker, and downregulate the expression of fibronectin, the mesenchymal cell marker. Also, the overexpression of TGF-*β*, T*β*R2, Smad3, and Snail were suppressed. Therefore, herbs-moxibustion combined with acupuncture can prevent intestinal epithelial-mesenchymal transition (EMT) in CD, and its mechanism is related to the TGF-*β*1-Smad-Snail pathway (Figure 1).

### 3.2. Toll-Like Receptors

Toll-like receptors (TLRs) are a family of pattern recognition receptors, and the innate immune system disorders mediated by them are the core participants in the pathogenesis of IBD. Toll-like receptor 4 (TLR4) is a receptor on the surface of immune cells, which plays a significant role in body immunity. It can activate the myeloid differentiation factor 88 (MyD88) signal transduction pathway and lead to nuclear translocation of nuclear factors-*κ*B (NF-*κ*B). NF-*κ*B is a key molecule that transduces intestinal inflammation, which regulates each other with inflammatory cytokines and amplifies the inflammatory response [27]. The TLR4/MyD88/NF-*κ*B signaling pathway has been abnormally activated throughout the process of intestinal mucosal damage, which is considered to be one of the targets of UC treatment [28, 29].

Qiao et al. [30] found that the levels of TLR4, MyD88, and NF-*κ*B in the colon of UC rats were significantly higher than those of normal rats, which confirmed the abnormal activation of the TLR4/MyD88/NF-*κ*B signaling pathway. However, after the intervention of electroacupuncture, the levels of TLR4, MyD88, and NF-*κ*B decreased, while the levels of IL-4 and IL-10 increased and IL-17 and PGE_2_ decreased, indicating that acupuncture can inhibit the activation of the TLR4/MyD88/NF-*κ*B signaling pathway, decrease the expression of proinflammatory factors, and increase anti-inflammatory factors, thereby reducing the intestinal inflammatory response. Li et al. [31] found electroacupuncture at Tianshu (ST25) could downregulate TLR4, MyD88, and NF-*κ*B p65 in the colon, IL-1*β* and TNF-*α* in serum, and upregulate IL-10 in serum, which was more effective than sulphasalazine. The expression of p-I*κ*B*α* and p-p65 can also be inhibited via manual acupuncture and electroacupuncture, which indicates that acupuncture can inhibit the activation of NF-*κ*B [32] (Figure 1).

### 3.3. NLRP3 Inflammasomes

NOD-like receptor protein 3 (NLRP3) inflammasomes are protein complex in the cytoplasm, which consists of NLRP3, apoptosis-associated spotted protein (ASC), and caspase-1 precursor protein. NLRP3 inflammasomes are crucial for innate immunity and contribute to inflammatory diseases such as IBD [33, 34]. Seo et al. [35] demonstrated in the DSS-induced mice model that the activation of NLRP3 inflammasome was involved in colitis. The activation of NLRP3 can activate macrophages to secrete IL-1*β* [36]. By inhibiting the activation of NLRP3 in macrophages, the experimental colitis can be improved [37]. Electroacupuncture at ST36 could inhibit the activation of NLRP3 and caspase-1 and reduce the level of IL-1*β* in macrophages in DSS-induced mice. In the meanwhile, the percentage of M1 macrophages increased and M2 macrophages decreased, which was reversed by electroacupuncture. The results indicated that electroacupuncture may ameliorate colitis by suppressing the NLRP3/IL-1*β* pathway [38]. Furthermore, the production of reactive oxygen species (ROS) is the main activation mechanism of the NLRP3 inflammasome, which can lead to the activation of the NLRP3 inflammasome and the release of inflammatory factors [39]. NADPH oxidases (NOXs) are rapid reaction enzymes that produce ROS. When activated, it will catalyze the production of ROS, which constitutes the molecular basis of oxidative stress [40]. Zeng et al. [41] found that the expression of NOXs, ROS, NLRP3, and IL-1*β* increased in TNBS-induced rats compared with those in normal rats but significantly decreased after 14 days of electroacupuncture treatment, which proved that electroacupuncture may treat UC by affecting the NOXs-ROS-NLRP3 signaling pathway (Figure 1).

### 3.4. Oxidative Stress and Endoplasmic Reticulum Stress

Oxidative stress is involved in the pathogenesis and progression of IBD [42, 43]. It is found that the decreased ability of the body to scavenge oxygen free radicals is one of the factors that cause inflammation and aggravate ulcers [44]. MDA is a marker of oxidative stress. SOD has the physiological function of scavenging oxygen free radicals. In the DSS-induced UC model, MDA activity increases and SOD activity decreases [45]. Endoplasmic reticulum stress (ERS) plays a key role in the occurrence and development of IBD because it relates to the persistence of inflammatory and autoimmunity [46, 47]. Wu et al. [32] demonstrated that both manual acupuncture and electroacupuncture could upregulate SOD and CAT and downregulate MDA in UC rats, which indicated that acupuncture can suppress oxidative stress induced by TNBS. In the meanwhile, the levels of GRP78, p-PERK, and p-eIF2*α* decreased, indicating that acupuncture can inhibit ERS (Figure 1).

## 4. Acupuncture Exerts Immunomodulatory Effects by Regulating Adaptive Immune System

### 4.1. The Balance of Th17/Treg Cells

Th17 cells are involved in the occurrence and development of inflammatory diseases and autoimmune diseases and are the main participants in IBD. Treg cells, the immunomodulatory cells maintaining immune tolerance, can inhibit intestinal inflammation [48]. It was found that Th17 cells in the peripheral blood and intestinal mucosa of IBD patients were significantly higher than those of healthy people, while Treg cells were significantly lower [49]. Th17 cells can secrete cytokines such as IL-17, IL-21, and IL-22. When the body is in a normal state, Th17 cells and their secreted cytokines can resist pathogen infection in vitro, thereby maintaining intestinal immune homeostasis. When the body is stimulated by antigen, the initial CD4^+^T cells differentiate, leading to the disorder of Th17 cells regulation and inducing an abnormal immune response. Treg cells play an immunosuppressive role by secreting inhibitory cytokines such as IL-10 and TGF-*β* [50].

#### 4.1.1. The Ratio of Th17 and Treg Cells

Th17 and Treg cells restrict each other to maintain the balance of the immune system [51]. Once the balance is broken, it will cause a variety of autoimmune diseases and intestinal inflammatory responses [52]. The imbalance between the Th17 and Treg cells has been shown to be an important cause of IBD [53]. Adjusting the balance between the two can directly regulate the expression of proinflammatory and anti-inflammatory factors, which can help improve intestinal inflammatory response and rebuild intestinal immune balance [54].

Studies have shown that the number of Treg cells in the mice model was downregulated whereas Th17 cells were upregulated [55]. However, electroacupuncture can upregulate the CD4^+^CD25^+^Foxp3^+^Treg cells and downregulate the CD3^+^CD8^+^IL-17^+^Th17 cells in spleen lymphocytes of UC mice [56] so as to improve the ratio of Treg and Th17 cells. The results of Sun et al. also proved this point [57].

#### 4.1.2. Proinflammatory Cytokines

Cytokines are secreted by immune cells [58], such as lymphocytes and macrophages, which can be classified into proinflammatory cytokines and anti-inflammatory cytokines. The balance of two cytokines is essential for maintaining intestinal immune homeostasis. Th17 cells secrete cytokines, such as IL-17, IL-21, and IL-22, which can induce and aggravate inflammatory responses. IL-17 is a hallmark cytokine of Th17 cells, which can induce inflammatory response [59]. IL-17 mRNA and protein levels in the blood of IBD patients are significantly upregulated [49]. IL-22 can maintain the integrity of the epithelial barrier and protect mucin-secreting goblet cells [60]. IL-23 is mainly produced by macrophages, and its overexpression in intestinal mucosa will destroy the defense barrier and affect immune regulation [61]. Existing studies have proven that Th17/IL-23 immune axis is the main immune response pathway in the pathogenesis of CD and plays a key role in intestinal inflammation [62, 63]. IL-6 is the key factor in determining whether the initial CD4^+^T cells differentiate into Treg cells or Th17 cells. Blocking the IL-6 signaling pathway can inhibit the differentiation of Th17 cells [64].

Liang [65] used warm needle acupuncture to treat patients with UC and found that the levels of IL-17 and IL-23 in serum decreased significantly after two weeks. Chen [66] demonstrated that electroacupuncture at Tianshu (ST25) and Zusanli (ST36) can reduce the contents of IL-6 and IL-17 in the serum of UC rats. In order to observe the effects of moxibustion and acupuncture on the expression of IL-17A and IL-22 in Crohn's disease rats, Liu [67] established the Crohn's disease model with 2,4,6-trinitrobenzene sulfonic acid (TNBS). After 15 minutes of intervention with moxibustion and acupuncture at Tianshu (ST25) and Shangjuxu (ST37), respectively, the results showed that compared with the model group, the expression of IL-17A decreased in the moxibustion group, while there was no change in IL-22. However, there was no difference between IL-17A and IL-22 in the acupuncture group. It is suggested that acupuncture may not inhibit the expression of IL-17A and IL-22 in Crohn's disease model rats.

#### 4.1.3. Anti-Inflammatory Cytokines

Treg cells secrete anti-inflammatory cytokines (IL-10, TGF-*β*, etc.) that can inhibit intestinal inflammation. IL-10 plays an immunomodulatory role in many ways. For example, it can maintain intestinal immune homeostasis by inhibiting the release of TNF-*α* [68]. It can also inhibit the proliferation of Th cells and reduce the secretion of harmful cytokines. Studies have shown that IL-10 deficiency can lead to somatic mutation and increase the risk of carcinogenesis in the IBD model [69]. In addition to determining the differentiation of CD4^+^ cells in collaboration with IL-6, TGF-*β* also promotes epithelial wound healing and tissue repair [70]. It was found that reduced TGF-*β* signal transduction in T cells and dendritic cells led to colitis in model mice [71], and TGF-*β*-deficient colonic epithelial cells and lamina propria showed inflammatory damage. Moreover, TGF-*β* can be used as an anti-inflammatory cytokine for the treatment of IBD. Zorzi et al. [72] found that TGF-*β*_1_ can improve the fibrosis of IBD.

The study of Chen [66] showed that the level of IL-10 in serum and the positive cells of TGF-*β* in the colonic mucosa of UC rats decreased significantly. However, the levels of the two increased significantly after electroacupuncture intervention. Electroacupuncture can also elevate the expression of TGF-*β*, IL-10, and IL-2 in dextran sulfate sodium (DSS)-induced UC mice [57]. Studies also showed that the contents of IL-6 and TGF-*β* in the serum of DSS mice increased significantly, while electroacupuncture can inhibit the elevation [73].

#### 4.1.4. Transcription Factors

Foxp3 and ROR*γ*t determine the direction of T cell differentiation. Foxp3, a member of the fork-head transcription factor family, is a marker transcription factor of Treg cells. It can affect the growth and development of Treg cells [74]. Mutations in Foxp3 lead to autoimmune diseases, and defects in Foxp3 lead to intestinal mucosal inflammation. ROR*γ*t is a key transcription factor for differentiation of Th17 cells. Inhibition of its expression can directly inhibit the differentiation of Th17 cells and reduce the level of Th17 cells, thereby reducing the inflammatory response [75]. When the body is under normal conditions, the two are in dynamic balance. When stimulated, the expression of Foxp3 is downregulated or ROR*γ*t is upregulated, which promotes the differentiation of Th17 cells and the release of inflammatory factors. In contrast, Treg cells differentiate and play an anti-inflammatory role [76]. Therefore, the balance between ROR*γ*t and Foxp3 is the key in determing the balance of Th17/Treg cells and regulating the immune state of the body. Sun et al. [57] found that the expression of ROR*γ*t increased and Foxp3 decreased in the colon of DSS-induced UC mice. After the intervention of electroacupuncture and moxibustion, the expression of ROR*γ*t was downregulated and Foxp3 was upregulated. Chen [66] demonstrated that electroacupuncture at Tianshu (ST25) and Zusanli (ST36) can increase the contents of Foxp3 and DAF in the intestinal tissue. Decay-accelerating factor (DAF) is a cell regulatory factor that regulates T cell response [77]. Signal transducers and activators of transcription 3 (STAT3), a key target for alleviating inflammatory response, can regulate the expression of IL-17 and promote Th17 differentiation [78, 79]. Hypoxia-inducible factor 1*α* (HIF-1*α*) can inhibit Treg cell differentiation by promoting Foxp3 degradation [80]. Both STAT3 and HIF-1*α* act on ROR*γ*t and Foxp3. Acupuncture can effectively reduce the expression of STAT3 and HIF1-*α* protein in the colon of UC mice induced by DSS [81, 82].

#### 4.1.5. CD39 and CD73

CD39 and CD73 are special markers on the surface of Treg cells. In UC mice, CD39-deficient Treg cells failed to exert immunosuppressive effects, and CD73-deficient Treg cells failed to produce extracellular adenosine [83, 84]. The CD39/CD73/A2a adenosine metabolic pathway plays an important role in the immune tolerance function of Treg cells. Studies have shown that patients with UC have lower adenosine levels, and the use of adenosine receptor A2a agonist can effectively reduce colitis and inhibit the production of proinflammatory factors [85, 86]. Zhuang et al. [87] found that the fluorescence intensity, the number of positive cells, and protein expression of CD39, DC73, and A2a in the colon of DSS mice increased after acupuncture. In the meanwhile, the ratio of CD39- and CD73-positive Treg cells in the peripheral blood, inguinal drainage lymph nodes, and spleen increased, indicating that the effect of electroacupuncture on UC mice may be related to the regulation of the CD39/CD73/A2a adenosine metabolic pathway and the influence of the anti-inflammatory effect of Treg cells.

### 4.2. The Balance of Th1/Th2 Cells

Th1 cells mediate cellular immunity and promote inflammation, mainly secreting IL-2, IFN-*γ*, and TNF-*α*. Th2 cells mediate humoral immunity and suppress inflammation, mainly secreting IL-4 and IL-10 [88].

To explore the effect of acupuncture and moxibustion on Th1/Th2 immune balance in UC rats, Chen [89] found that after the 14^th^ intervention of electroacupuncture at Tianshu (ST 25) and Qihai(CV6), the ratio of CD4^+^IFN-*γ*^+^/CD4^+^IL-4^+^ cells in the model group was higher than that in the control group, and the ratio in the electroacupuncture group was lower than that in the model group; the levels of IFN-*γ* and IL-12 in the electroacupuncture group were lower than those in the model group, and IL-4 and IL-10 were higher, which proved that acupuncture and moxibustion can regulate the balance of Th1/Th2 cells.

## 5. Acupuncture Exerts an Immunomodulatory Effects by Regulating Intestinal Flora

Intestinal flora plays a crucial role in the maintenance of physical health and the pathogenesis of gastrointestinal diseases [90]. Some studies have shown that the composition and number of the intestinal flora of IBD patients are lower than those of healthy people [91–93]. Intestinal flora can regulate immune cells in a certain way and then induce immune-inflammatory response. The imbalance of intestinal flora can affect the balance of Th17/Treg differentiation in certain ways, resulting in abnormal secretion of related inflammatory factors and intestinal inflammation, which is vital in the pathogenesis of IBD [94, 95]. In order to explore whether acupuncture and moxibustion can improve UC symptoms by regulating the intestinal flora and whether the diversity of intestinal flora is related to Treg and Th17 cells, Wei et al. [96] detected the genome of intestinal flora through Illumina-MiSeq sequencing. The results showed that electroacupuncture and moxibustion can improve the alpha diversity indices and beta diversity of the intestinal flora and inhibit *Streptococcus*, *Odoribacter*, and *Allobaculum* but facilitate *Lactobacillus*. Also, the correlation analysis showed that the increase in the abundance and diversity of the intestinal flora was positively correlated with Treg cells and negatively correlated with Th17 cells. It can also increase the content of *Lactobacillus* and *Spirillum* and reduce the content of *Clostridium* bicarbonate in UC model rats [97].

Gut microbiota is also closely related to the intestinal barrier [98]. Wang et al. [99] found that electroacupuncture can upregulate Bacteroidetes, Muribaculaceae, *Faecalibacterium*, *Roseburia*, and Bifidobacterium, while downregulating Firmicutes, Proteobacteria, *Escherichia-Shigella*, and *Erysipelatoclostridium*. To further confirm the influence of gut microbiota on the barrier protective effect of electroacupuncture, a fecal microbiota transplantation (FMT) experiment was used. Compared with DSS mice, mice that received microbiota from electroacupuncture have less colonic inflammation and better barrier integrity, which indicates that electroacupuncture maintains the integrity of the intestinal barrier by modulating the gut microbiota. Electroacupuncture can modulate the overall structure and structural segregation of the gut microbiota, specifically in the downregulation of Turicibacteraceae, Clostridiaceae, and Erysipelotrichaceae and upregulation of Lactobacillaceae [100].

## 6. Conclusion

By reviewing the existing literature, we found that the immunomodulatory mechanism of acupuncture in the treatment of IBD has the following characteristics: First of all, from the perspective of intervention methods, the studies of manual acupuncture and electroacupuncture have been the most studied, especially electroacupuncture. Although warm needle acupuncture and fire acupuncture are effective, their mechanisms are rarely studied. Then, from the perspective of research direction, the current research on immune regulation mainly focuses on adaptive immunity, especially on the balance of Th17/Treg axis, which may be due to the current recognition and clarity of the mechanism. The mechanism of innate immunity is insufficiently studied, such as oxidative stress and endoplasmic reticulum stress. In terms of the research results, the mechanism underlying the immune regulation of acupuncture involves innate immunity (intestinal epithelial barrier, toll-like receptors, NLRP3 inflammasomes, oxidative stress, and endoplasmic reticulum stress) and adaptive immunity (the balance of Th17/Treg and Th1/Th2 cells) as well as the intestinal flora. Although the current research has made certain progress, the explanation of the immune regulation of acupuncture is far from enough, and further research is needed.
